# Working memory training for adult hearing aid users: study protocol for a double-blind randomized active controlled trial

**DOI:** 10.1186/1745-6215-14-417

**Published:** 2013-12-05

**Authors:** Helen Henshaw, Melanie A Ferguson

**Affiliations:** 1 NIHR Nottingham Hearing Biomedical Research Unit, School of Medicine, University of Nottingham, Ropewalk House, 113 The Ropewalk, Nottingham, UK; 2 NIHR Nottingham Hearing Biomedical Research Unit, Nottingham University Hospitals NHS Trust, Ropewalk House, 113 The Ropewalk, Nottingham, UK

**Keywords:** Hearing loss, Hearing aid, Speech intelligibility, Speech perception, Cognition, Cognitive training, Working memory, Working memory training

## Abstract

**Background:**

One in ten people aged between 55 to 74 years have a significant hearing impairment in their better hearing ear (as defined by audiometric hearing thresholds). However, it is becoming increasingly clear that the challenges faced by older listeners cannot be explained by the audiogram alone. The ability for people with hearing loss to use cognition to support speech perception allows for compensation of the degraded auditory input. This in turn offers promise for new cognitive-based rehabilitative interventions. Working memory is known to be highly associated with language comprehension and recent evidence has shown significant generalization of learning from trained working memory tasks to improvements in sentence-repetition skills of children with severe to profound hearing loss. This evidence offers support for further investigation into the potential benefits of working memory training to improve speech perception abilities in other hearing impaired populations.

**Methods/Design:**

This is a double-blind randomized active controlled trial aiming to assess whether a program of working memory training results in improvements in untrained measures of cognition, speech perception and self-reported hearing abilities in adult hearing aid users (aged 50 to 74 years) with mild-to-moderate hearing loss, compared with an active control group who receive a placebo version of the working memory training program.

**Discussion:**

The present study aims to generate high-quality preliminary evidence for the efficacy of working memory training for adults with mild-to-moderate sensorineural hearing loss who are existing hearing aid users. This trial addresses a number of gaps in the published literature assessing training interventions for people with hearing loss, and in the general literature surrounding working memory training, such as the inclusion of an active control group, participant and tester blinding, and increased transparency in reporting.

**Trial registration:**

ClinicalTrials.gov identifier: NCT01892007. Date of registration: 27 June 2013.

## Background

More than one in ten people aged between 55 to 74 years have a significant hearing impairment (greater than 25 dB hearing loss (dB HL) averaged across 0.5 to 4 k Hz) in their better hearing ear [1]. Although a relationship between auditory and cognitive processing has been recognized for decades [2], it has become clear in recent years that the challenges faced by older listeners cannot be explained by the audiogram alone [3]. Difficulties faced by adults with hearing loss, particularly in complex and noisy environments, have led to a recent resurgence in interest in the link between audition and cognition [4-6].

Auditory training has been used for many years as a rehabilitative intervention for people with hearing loss. It can be defined as a process that involves teaching the brain to listen through active engagement with sound. Auditory training has been shown to result in improved performance for the trained task, for example, frequency discrimination in normally hearing adults and children [7,8]. However, for auditory training to be an effective intervention for those with hearing loss, any improvements shown for trained tasks need to generalize to functional benefits to real-world listening [9]. It has previously been suggested that for language and listening abilities, the development of cognitive skills may be equally, or perhaps even more important, than the refinement of sensory processing [10]. A recent study of auditory-cognitive training for older adults with hearing loss showed generalized improvements in neural timing, memory, speed of processing and speech-in-noise perception [11]. Research from our own laboratory suggests that auditory training using phonemic contrasts results in significant improvements in untrained measures of divided attention and working memory. Participants also reported post-training improvements in self-reported hearing abilities, specifically for a complex listening condition, ‘having a conversation with several people in a group’ [12]. Thus, training interventions that aim to improve cognitive performance may offer benefits to the listening abilities of people with hearing loss.

Working memory has been defined in many ways. Engle and Kane [13] emphasize the role of inhibition, suppressing interference from irrelevant sources of information. Barrouillet *et al*. [14] focus on resource sharing and the capacity to divide or switch attention. Miyake *et al*. [15] state that working memory offers a means to update and maintain information. In a review of a number of different models, Miyake and Shah concluded that working memory could be generally defined as those ‘mechanisms or processes that are involved in the control, regulation, and active maintenance of task-relevant information in the service of complex cognition, including novel as well as familiar, skilled tasks’ [16]. Working memory is known to be highly associated with language comprehension [17,18] and the neural processing of sound [19]. Working memory has also been recognized as an important predictor of an individual’s success with hearing aids [20,21]. Difficulties in hearing may be exacerbated by, or ‘masquerade as’ , reductions in cognitive performance, for example, problems in remembering or comprehending spoken language [22,23]. It has been suggested that the ability for people with hearing loss to use cognitive abilities (such as attention and working memory) to support context in speech perception allows for compensation of a degraded auditory input, which in turn offers promise for new cognitive-based rehabilitative interventions [24,25]. However, a recent systematic review of studies assessing the efficacy of *auditory* training for adults with hearing loss suggests a number of methodological and reporting inadequacies in published research, resulting in very-low to moderate quality evidence [9]. Thus, to draw adequate conclusions about the efficacy of training interventions (auditory or cognitive) for people with hearing loss, any future investigations should offer a high level of evidence using well-designed randomized controlled trials with ample transparency in reporting.

Cogmed RM is a training software product for children or adults that aims to improve working memory, comprising verbal and visuospatial working memory and memory storage tasks. Brehmer *et al*. [26] demonstrated significant improvements in performance for trained Cogmed tasks in younger (20 to 30 years old) and older (60 to 70 years old) adults. In addition, generalization of on-task learning was shown to improvements in untrained measures of sustained attention and self-reported cognitive function, and these improvements were maintained across a 3-month follow-up interval. A pilot study of Cogmed as an intervention for hearing loss [27] showed significant generalization of on-task learning to improvements in sentence-repetition skills of children with severe to profound hearing loss who were cochlear implant users.

There are currently 10 million people in the UK with a significant hearing impairment [28], the majority of whom have mild and moderate hearing loss. The most common management strategy for those individuals is the provision of hearing aids to amplify sound. However, additional support and interventions can improve outcomes for aided hearing impaired listeners [24]. This preliminary evidence [27] offers support for further investigation into the potential benefits of working memory training to improve listening and speech perception abilities for adult hearing aid users with mild to moderate hearing loss.

The present study protocol presents novel independent research funded by the National Institute for Health Research (NIHR) and has been reported in accordance with the SPIRIT (‘Standard Protocol Items: Recommendations for Intervention Trials’) 2013 guidance for content of a clinical trial protocol [29].

### Research objective

Does working memory training result in improvements in cognition, speech perception and self-reported hearing abilities in adults with mild-to-moderate hearing loss who are existing hearing aid users, compared with an active control group (non-adaptive, placebo training)?

### Specific research hypotheses

We **hypothesise** that:

1. Compared with the active control group, hearing aid users in the experimental group (adaptive working memory training) will have significant generalized improvements in untrained measures of cognition, speech perception and self-reported hearing abilities. Furthermore, these improvements will be significantly greater than for hearing aid users in the active control group who receive non-adaptive training.

## Methods/Design

**Figure 1:**
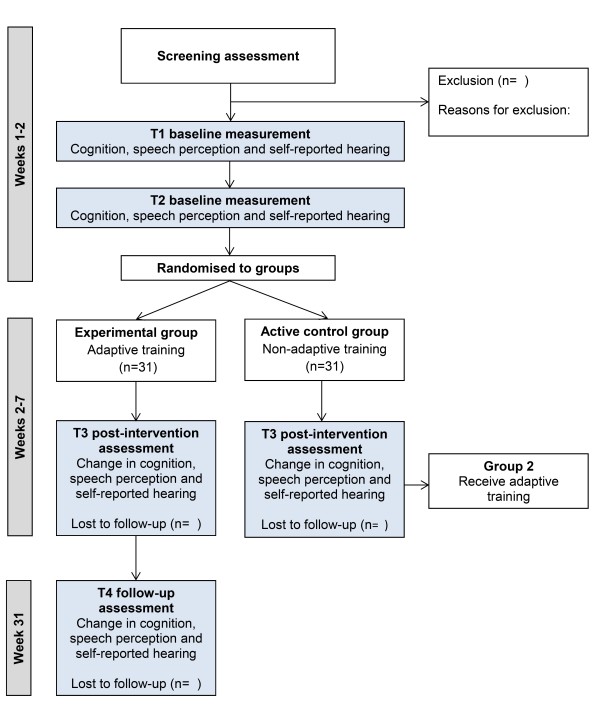
Study design.

The study is a single-center, phase II, double-blind, randomized, active controlled trial, with minimized allocation of participants to one of two groups (Figure 1). One group (experimental) will receive adaptive working memory training, where task difficulty adjusts according to individual participant performance. The second group (active control) will receive non-adaptive training, fixed at low-difficulty practice level (3 to-be-remembered items). The use of an active control group for comparison will help account for any placebo effects, thus increasing confidence in the estimation of effect in the adaptively trained group that improvements in outcomes result from the development of working memory ability (as trained using an adaptive training program), rather than simply the provision of a the training program *per se*. The research edition of Cogmed RM working memory training will be used as this has been designed for double-blind randomized active controlled trials and provides no indication to either participants or testers of which training group (experimental or active control) a participant has been allocated.

One initial assessment and two baseline sessions, conducted a maximum of 1 week apart (T1 and T2), will be completed prior to allocation of training. The two baseline sessions will help account for any improvements in outcomes as a result of test-retest effects (procedural learning) prior to the intervention, thus increasing our confidence in the estimation of subsequent intervention-specific effects.

Working memory training will be delivered in participants’ homes via an online training portal. Participants will complete a total of 25 sessions (5 sessions per week for 5 weeks). Improvements for trained tasks will be assessed using the Cogmed ‘index of improvement’. Following training, participants will return for a post-intervention outcome assessment at T3. Participants in the active control group who receive the non-adaptive training will be unblinded following their T3 assessment and offered the adaptive training. Participants in the experimental group will remain blinded at T3 and will be invited to return for a 6-month follow-up assessment at T4 (31 weeks) to assess retention of any post-training improvements in outcomes (Figure 1).

The study has obtained approval from the Nottingham University Hospitals NHS Trust Research and Innovation Department, the trial sponsor (study reference 08ET002-01), and full ethical approval from Nottingham 2 NHS Research Ethics Committee (reference 08/H0408/172).

### Participant sample

Existing hearing aid users between the ages of 50 to 74 years with mild to moderate hearing loss, as defined by an audiometric air-conduction pure-tone averaged (averaged instead of average) (PTA) across octave frequencies between 0.25 to 4 kHz of 20 to 70 dB HL [30] in the better hearing ear, will be recruited from the NIHR Nottingham Hearing Biomedical Research Unit database of research volunteers in the UK.

#### Inclusion criteria

Inclusion criteria are: (1) aged 50 to 74 years; (2) existing (3+ months) hearing aid(s) user; (3) mild to moderate (PTA_0.25-4k Hz_ 21 to 69 dB HL) sensorineural hearing loss (SNHL; air bone gap average across 0.5, 1 and 2 kHz ≤ 15 dB HL) in the better hearing ear; and (4) internet access at home.

#### Exclusion criteria

Exclusion criteria are: (1) having previously taken part in a training intervention study; (2) first language other than English (all speech outcome measure are presented in English); (3) unable to use a desktop or laptop computer (as working memory training is delivered via the internet using a desktop or laptop computer); and (4) mild cognitive impairment as defined (as a score of less than 26/30 on the Montreal Cognitive Assessment [31]).

### Outcome measures and power analysis

#### Primary outcome measure

Change in performance in an untrained measure of verbal working memory (Visual Letter Monitoring Task) will be the primary measure of efficacy in the present study. The Visual Letter Monitoring Task [32] is a visual measure of verbal working memory updating that is not trained within the Cogmed package. There are 10 consonant-vowel-consonant (CVC) words embedded in an 80-letter sequence. Two sequences will be presented to participants at each visit in counterbalanced order. Individual letters are displayed sequentially on a computer screen at a rate of 2 s per letter (first list) and 1 s per letter (second list). Participants will be asked to press the keyboard ‘space bar’ (hit) when three consecutive letters formed a recognized CVC word (for example, M-A-T). The test provides two measures, total number of hits (maximum score of ten per list) and d’ (d prime).

#### Secondary outcome measures

Secondary outcome measures assess multiple aspects of speech perception, cognition and communication abilities.

### Speech perception

#### Phoneme discrimination

The phoneme discrimination task assesses an individual’s ability to distinguish differences between phonemes presented on a continuum. Delivered using the Medical Research Council (MRC) Institute of Hearing Research System for Testing Auditory Responses (IHR-STAR) platform. Participants will be presented with three discrete phonemes from a continuum per trial and asked to identify the odd one out. Phoneme continua /a/ /e/ (easy) and /d/ /g/ (difficult) will be presented for a block of 35 trials in sequential blocks, with a 3-trial demonstration of continuum /a/ /e/ prior to the 2 blocks. The task will be adaptive based on participant performance using a three-phase adaptive staircase procedure (see [33] for further information), with auditory and visual feedback provided to participants after each trial (correct/incorrect response). Threshold will be calculated as the average of the last 2 reversals in a block of 35 trials.

#### Context

High-predictability and low-predictability (context) sentences [34], based on the Revised Speech Perception in Noise Test [35], are produced by a British English native speaker. Lists of 22 sentences (11 high predictability and 11 low predictability) are presented in the free field in a background of multi-talker babble at a fixed signal-to-noise ratio (SNR) of -1 dB. Two practice sentences (one high and one low predictability) are presented to participants at a slightly more favorable (2 dB) SNR prior to commencing the main test. Participants are asked to listen to each sentence and repeat the last word aloud. The test is scored as the percentage of last words correctly repeated for both high-predictability and low-predictability lists.

#### Competing speech

The Modified Coordinate Response Measure (MCRM) is a measure of speech perception ability in the presence of different maskers at an adaptive signal-to-noise ratio. The basic task is described by Hazan and colleagues [36], and is based on the Coordinate Response Measure [37]. Participants will be presented with sentences in the form of ‘show the [animal] where the [color] [number] is’. There are six possible monosyllabic animals (cat, cow, dog, duck, pig and sheep), six colors (black, blue, green, pink, red and white) and eight numbers (one to nine, excluding multisyllabic seven). Two sentences are presented concurrently, one by a female speaker (target) and one by a male speaker (distracter). Participants will be asked to listen for the color and number spoken by the female speaker (‘dog’ is always the animal target) while ignoring the male speaker, and respond by pressing the corresponding target color number on a computer touchscreen. The test uses an adaptive 1-up 1-down staircase method with an initial step size of 10 dB until the first reversal, reducing to 7 dB at reversal 2 and 4 dB at reversal 3. The test continues until a total of eight reversals are achieved and the test is completed twice by each participant. Speech reception thresholds are calculated in this study using the average of the last two reversals, averaged across the two runs.

### Cognition

#### Simple-span working memory

The digit span (backwards) subtest from the Wechsler Adult Intelligence Scale, Third Edition (WAIS-III) [38] is measure of simple-span working memory that involves listening to a list of numbers of increasing length and repeating them in reverse order. Digits are presented at each list length twice and lists increase in length by one digit if participants correctly recall one of the two lists at each length correctly, otherwise the test is discontinued. The performance is scored as the total number of lists correctly repeated in reverse order. A version of the digit span (backwards) forms one of the trained tasks in the Cogmed RM program.

#### Auditory attention

The MRC Institute of Hearing Research Test of Attention in Listening (TAIL) is a measure of auditory attention [39] using tones that vary in both frequency and spatial location. Primary tasks include both frequency and location discrimination and participants are asked to respond as to whether two tones are the ‘same’ or ‘different’ frequency or location using a button box response. Tones are presented in the free field at participants’ most comfortable loudness (MCL) level [40] via two speakers situated at 90° to the left and 90° to the right of the participant. TAIL measures the ability to focus selectively on a task relevant dimension (either frequency or location) and ignore information from task irrelevant dimensions, using reaction time (RT) as the primary performance measure. The task is scored using measures of involuntary orientation (the impact of the task irrelevant dimension on RTs for the task relevant dimension, quantified as the difference in RTs between ‘same’ and ‘different’ trials) and conflict resolution (the frequency by location interaction, quantified as the difference between congruent (same or different in both dimensions) and incongruent (same in one dimension, different in the other) trials).

#### Single and divided attention

The Test of Everyday Attention (TEA) subtests 6 (telephone search) and 7 (telephone search while counting) will be used to assess participants single (visual) attention and dual (auditory and visual) attention [41]. In subtest 6, participants are asked to search a telephone directory for matching symbols. In subtest 7, participants are asked to search a telephone directory for matching symbols while counting strings of beeps in varying lengths (2 to 12) that are presented in the free field. The task is scored using time (in seconds) per correctly identified symbol for subtests 6 and 7, and weighted in subtest 7 by the proportion of correctly counted beep strings. A dual task decrement can be calculated, which provides the difference in time (in seconds) per correctly identified symbol where two simultaneous tasks are being completed, compared with that for a single task (subtest 7 minus subtest 6).

#### Working memory and inhibition

The Size Comparison Span (SICSPAN) is a measure of working memory capacity including inhibition of semantic confusions [42]. Participants view lists of size comparisons (for example, ‘tree is larger than acorn’ to which they must respond ‘yes’ or ‘no’ using a button box response. Participants are then provided with to-be-remembered words from the same semantic category (for example, ‘leaf’). At the end of the list, participants are required to recall the to-be-remembered words while inhibiting words included in the size comparison judgments. The task begins with lists of two size comparison judgments and to-be-remembered words, increasing to list lengths of three, four, five and six. There are two trials at each list length. The task continues until all list lengths have been presented, with no discontinuation rule.

#### Dual task of listening and memory

The dual task is a measure of listening and memory designed to assess listening effort [43]. Participants are presented with a five-digit memory task that flanks a speech in noise comprehension task. A string of five digits are displayed visually on a computer screen for 5 s. Participants are asked to retain the digits in memory for later recall. Participants are then presented with a list of five AB isophonemic monosyllabic words [44] presented in a background of multi-talker babble, and are asked to repeat each word immediately after presentation. After each list of five words, participants are asked to recall the previously presented five digits. There are 4 word lists, resulting in a maximum possible score of 20 correctly repeated words and 20 correctly recalled digits. A dual-task score is calculated by adding together the scores for the word and digit tasks, resulting in a maximum possible dual-task score of 40.

### Self-reported communication

The Glasgow Hearing Aid Benefit Profile [45] is used to assess self-reported hearing disability (activity limitation), hearing handicap (participation restriction), and hearing aid use, benefit, and satisfaction. This questionnaire will be administered via interview and completed electronically. Participants will be presented with series of four listening scenarios (listening to the television, having a conversation with one other person in a quiet room, having a conversation in a busy street or shop, talking to several people in a group) and are asked to rate the amount difficulty they have in the situation while wearing their hearing aids (initial disability/activity limitation, 1 = no difficulty to 5 = cannot manage at all) and how much any difficulty worries, annoys or upsets them (handicap/participation limitation, 1 = not at all to 5 = very much indeed). Participants then rate their hearing aid use (1 = never to 5 = all the time), benefit (1 = no use at all to 5 = hearing is perfect with aid) and satisfaction with their hearing aids (1 = not satisfied at all to 5 = delighted with aid). The mean of all four scenarios in each measure (Initial Disability, Handicap, Hearing Aid Use, Hearing Aid Benefit and Hearing Aid Satisfaction) are converted to a percentage (0% to 100%).

The Hearing Handicap Inventory for the Elderly [46] is a self-report questionnaire that quantifies the emotional and social/situational effects of self-perceived hearing impairment. Participants are asked to complete the 25-item paper questionnaire answering statements such as ‘Does a hearing problem cause you to be nervous’ with either ‘yes’ (4 points), ‘no’ (0 points) or ‘sometimes’ (2 points). The questionnaire is scored as total points for all items (maximum 100 points). Subtotal scores can also be calculated for emotional (12 items, maximum 48 points) and situational items (13 items, maximum 52 points).

### Power analysis

In order to detect a minimum improvement of 1.5 words (15%) in the primary outcome measure (Visual Letter Monitoring Task) based on 80% power, a 1-sided type I error rate of 5%, using a pooled standard deviation of 2.118 to derive the effect sizes, a total of 27 participants are required in each training group. We anticipate a participant attrition rate of no more than 15%, and therefore will recruit a total of 31 participants per group.

### Test procedure

Participants will be sent a study information sheet at least 24 h before their first test session. At the first test session, participants will provide signed informed consent. All testing will be carried out at the NIHR Nottingham Hearing Biomedical Research Unit. Audiometric testing will be performed in double walled sound attenuating booth. Speech perception and cognitive testing will take place in a quiet, purpose-designed test room. Auditory stimuli will be presented in the free field via a single speaker (Genelec Inc., MA) situated directly in front of the participant at their most comfortable loudness (MCL) level [40].

Cogmed RM working memory training will be delivered via the internet at the participant’s home, using either their home PC or a laptop loaned by the NIHR Nottingham Hearing Biomedical Research Unit. Auditory elements will be presented in the free field using built-in or portable speakers at participants’ MCL. Training will be home-based with telephone monitoring once per week to ensure progress is maintained and to monitor for any practical or technical issues with training. Participants will be supported throughout their at-home training by a training aide, typically their spouse, who will offer support and guidance in accordance with Cogmed guidelines. Training aides will be instructed on their role by a qualified training coach at the NIHR Nottingham Hearing Biomedical Research Unit.

### Randomization and blinding

#### Allocation to training groups

Participants will be allocated either an ‘experimental’ or an ‘active control’ user ID to access the training URL by the chief investigator (HH) using Minim, a method of minimization [47]. Minimization will ensure balanced allocation to the experimental and active control groups based on participants’ age (younger = 50 to 62 years vs older = 63 to 74 years), sex (male, female), hearing amplification type (unilateral vs bilateral hearing aids) and low versus high working memory capacity (backwards digit span score <7 vs ≥7) at baseline. Participant group allocation details will be documented and stored securely by the chief investigator in a password protected file.

#### Blinding of participants

Participants will be blind to group allocation and it will not be possible for participants to tell from the online training interface which group they have been assigned to.

#### Blinding of tester

The researcher responsible for testing participants at baseline and post-intervention outcome assessments will not be involved in the allocation of participant IDs and will be blind to participants’ group allocation.

### Intervention

**Figure 2:**
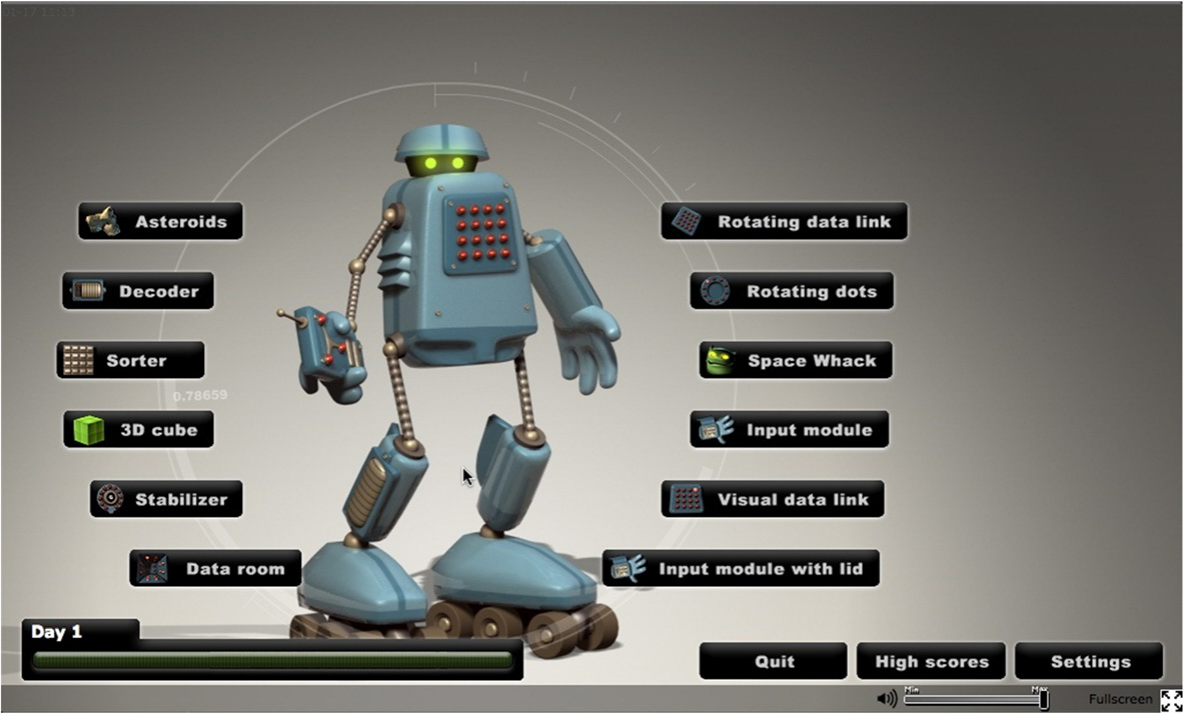
User home screen for working memory training.

Cogmed RM working memory training (Figure 2) consists of 11 different sequence-based verbal and visuospatial working memory and memory storage games (Table 1). Participants are required to complete 25 sessions over 5 weeks (5 sessions per week), actively training for 35 to 45 minutes a day.

**Table 1:** Working memory training tasks

**Training tasks**	**Task type**
Asteroid, sorter, 3D cube, rotating data link	Visuospatial working memory
Input module with lid	Verbal working memory
Input module, stabilizer	Visuospatial and verbal working memory
Data room, visual data link, space whack	Visuospatial storage
Decoder	Verbal storage

#### Adaptive training

For the experimental group, training task difficulty (number of to-be-remembered items) is adaptive, based on individual performance, to maintain average daily performance levels of approximately 60% of trials correct.

#### Non-adaptive training

For the active control group, non-adaptive training tasks are fixed at a low-difficulty practice level (three to-be-remembered items) and task difficulty will not increase for the duration of the training.

### Data management

The chief investigator is the data custodian. This study will abide by the Caldicott principles. Data will be stored in accordance with NHS guidelines.

Data collected on paper will be entered onto a database accessible only by the research team and stored alongside electronic data. The database will be password protected and held on the NIHR Nottingham Hearing Biomedical Research Unit network, which is also protected with a password that is changed every 12 months. Paper files will be stored in filing cabinets in a locked room. All identifiable files will be stored separately from data. Data will carry a unique (non-identifiable) study number. Electronic data is backed up daily, with backup files stored off site in a fireproof cabinet. Only the primary investigator, chief investigator, and the unit statistician will have access to the final data set.

### Data analysis and statistical methods

Analyses will be performed to include those of the 62 recruited participants who meet the study criteria and complete the trial. Any participant leaving the study before its completion will not be replaced. Missing data will be accounted for using a method of multiple imputation (replaced with plausible values based on Monte Carlo-simulated data points from the data set). Effect sizes will be calculated using Cohen’s *d*, using the pooled standard deviation of the two groups.

### Baseline characteristics

Baseline characteristics (including but not limited to; age, sex, hearing loss, working memory capacity, hearing aid use, hearing disability, hearing handicap, speech perception) will be described for each group.

### Efficacy analyses

The primary endpoint and first analysis of group data will take place at the end of the 7-week randomized controlled trial (week 7). Repeated measures analysis of variance (ANOVA) will be used to assess any main effects of time and training group (experimental vs. active control) on the primary and secondary outcome measures. In addition, exploration of any significant interaction effects will enable the identification of whether Cogmed (adaptive) training for participants in the experimental group results in significantly different outcome performance than Cogmed (fixed) training for participants in the active control group. The mean difference in the primary outcome measure (Visual Letter Monitoring Task) for the main intervention assessment comparison (T2-T3) will be presented as a mean difference and 95% confidence interval (CI) for each of the two groups. Within-group and between-group comparisons for the primary and secondary outcome measures will be conducted using paired and independent t tests (or non-parametric equivalents). Pearson’s product moment (or Spearman’s rho correlations) will be used to identify relationships between improvements in behavioral performance of speech perception and cognition and improvements in self-reported hearing ability, to help inform clinically significant benefits for people with hearing loss.

The second endpoint and planned analysis will take place at the end of T4 (week 31), for the experimental group only, to examine the retention of any post-training improvements in the primary and secondary outcomes at a 6-month follow-up assessment. Repeated measures ANOVA will be used to assess any main effects of time the primary and secondary outcome measures for participants in the experimental group. The mean difference in the primary outcome measure (Visual Letter Monitoring Task) for the main retention assessment comparison (T3-T4) will be presented as a mean difference and 95% confidence interval (CI). Within-group comparisons for the primary and secondary outcome measures will be conducted using paired and independent t tests (or non-parametric equivalents).

### Subgroup analyses

Repeated measures ANOVA, paired t tests and independent t tests (or their non-parametric equivalents) will be performed where appropriate to assess intervention efficacy in subgroups of participants who are bilateral and unilateral hearing aid users, and those with high and low baseline working memory capacity. Analyses will identify whether there are any differences in pre-intervention to post-intervention outcome performance (week 7), and for the retention of any training-related improvements (week 31), across different participant subgroups.

### Adverse events

It is not anticipated that there will be any adverse events in this trial. However, should any adverse events be identified, these will be recorded and reported according to the trial sponsor’s (Nottingham University Hospitals NHS Trust) Standard Operating Procedures.

## Discussion

Auditory training is currently used for the clinical management of people with hearing loss despite a lack of published high-quality evidence assessing its efficacy and mechanisms of benefit [9]. Benefits of auditory training to speech perception abilities of people with hearing loss may lie in bottom-up sensory refinement, top-down cognitive control, or a combination of the bottom-up and top-down improvements [11,12]. Although preliminary evidence exists regarding the efficacy of working memory training to improve sentence repetition skills for children with severe to profound hearing loss [27], this is yet to be examined in other hearing impaired populations.

The present study aims to generate high-quality preliminary evidence for the efficacy of working memory training to improve cognition, speech perception and self-reported hearing abilities in adults with mild-to-moderate sensorineural hearing loss who are existing hearing aid users. This randomized active controlled trial addresses a number of gaps in the current published literature [9], offering high-level evidence for the benefits to people with hearing loss that may be offered by a program of working memory training.

## Trial status

The trial is currently in the recruitment phase. It is expected that recruitment into the study will be complete by 31 January 2014.

## Abbreviations

dB: Decibel; GHABP: Glasgow hearing aid benefit profile; HHIE: Hearing handicap inventory for the elderly; HL: Hearing loss; MCRM: Modified coordinate response measure; MoCA: Montreal cognitive assessment; PTA: Pure tone average; SICSPAN: Size comparison span; STAR: MRC Institute of Hearing research System for Testing Auditory Responses; TAIL: Test of attention in Listening; TEA: Test of everyday attention.

## Competing interests

The authors declare they have no competing interests. Manuscripts reporting study results will be reviewed by Cogmed representatives 30 days prior to submission to a peer-reviewed journal. However, the NIHR Nottingham Hearing Biomedical Research Unit has the right to publish all statistical analyses regardless of positive or negative results.

## Authors’ contributions

HH and MAF developed the protocol. HH drafted the manuscript. Both authors contributed to editing and approved the final manuscript. The views expressed are those of the authors and not necessarily those of the NHS, the NIHR, or the Department of Health.
